# Employment for Epileptics

**Published:** 1887-02-19

**Authors:** Nina Paget


### Employment for Epileptics.

By Miss Nina Paget.

Special Difficulties.

Of all the ills to which flesh is heir, none claims our pity more than the mysterious disease epilepsy. Many hospitals for other diseases stand open, and sooner or later the sufferers are cured by medical skill, or released by death. With the epileptic it is different. He is apparently strong, healthy, capable of work, and is anxious to do it. No sign perceptible to the inexperienced eye marks him out from others. But, at any moment, without an instant's warning, the fit may seize him; at the top of a scaffold or staircase, by the side of a river, or near the red-hot bars of a furnace. With a scream that echoes as one of the most awful sounds that penetrates human ears, this hearty, healthy man falls absolutely unconscious, writhing, foaming at the mouth, a sight never to be forgotten. In a short time the patient wakes up again, restored to apparent health, and often entirely unaware that anything more than a slight temporary faintness or unconsciousness has taken him—unless, as sometimes happens, terrible burns or bruises remain to show where and how the fall had taken place. The absence in most cases of any warning, and this curious unconsciousness, make epileptics very hard cases to deal with, and a cause of great anxiety to those responsible for them. These circumstances also make the question of any employment for them most difficult. It is evident that the avocations of daily life in the usual routine are quite unfitted for them. Who would employ them, for instance, in a mill among machinery where any fall must be horrible, in any workshop with unguarded staircases, and tools of all descriptions lying about?

Worst for Women.

If it is hard for men to find suitable employments, it is still worse for women. Teaching, the care of children, domestic service, the numerous workshops where women, married and unmarried, are employed, are all closed to them. Even if raised by circumstances above absolute want, how dreary must be their lives! Church, the theatre, the concert-hall, all places of public resort, are forbidden. This is sad, but the worst remains to be told. If work is found, and the disease is discovered by the employer, and the worker is dismissed; if the parent dies or becomes unable to support his child; what resource is left open? To these respectable, sober men and women, able and willing to work, there is nothing but the wards of the workhouse, unless wealth enables them to pay the necessarily high fees of a physician's private asylum. To become *paupers* during years of comparative health, may be the lot of these respectable and often well-born persons.

The French System.

Some time ago the Charity Organisation Society invited several members of the medical profession, whose positions as heads of large asylums and other institutions for the care and cure of epilepsy and insanity rendered their opinion of great weight, to meet them and give their experience as to the possibility of profitable employment for epileptics, and the callings most suitable for them to follow. Some Poor-law Guardians were also present at the meeting, one of whom, Miss Davenport-Hill, read parts of an interesting letter from Dr. Rolland, of the "*Asiles John Bost*," France, which we give here. Dr. Rolland says:

"By the side of the distinctively medical treatment, whether hydropathic or by bromides, or by both methods combined, and upon the same level, I think we must place the social treatment: I mean that which gives a fixed and regular occupation to the epileptic patient, and which affords him, in certain circumstances, an opportunity of appearing in the world outside the walls of the asylum to take a place there, certainly obscure and modest, but which allows him to live at liberty, and be no longer a burden on his family or to society at large. It is to this end, and to distract him from the evil suggestions which idleness and inaction fatally engender, that the doctor, and those who have specially occupied themselves with this subject of epilepsy, have created and annexed to the epileptic asylums, primary schools and technical workshops. In the classes, which should not last more than half an hour, the child will develop his intelligence, which has been enfeebled or retarded by the disease, he will learn the elementary notions which are indispensable to every citizen of however moderate capacity. In his workshop he will find a pleasant change from his work in school. Work suited to his strength and intelligence will powerfully assist him in physical development and the amelioration of his disease. But what occupation is given him? What pursuit can be taught him? The noblest of occupations—and that least followed by the great mass of people healthy in body and mind—namely, agriculture, the cultivation of the earth, is that which seems to me best fitted to the needs and exigencies of the social condition of most of those epileptics who people the special asylums. At Laforce, a somewhat important agricultural department is annexed to the asylum for epileptic men. A certain number of the patients are occupied every day in the various labours of the fields and gardens, under the superintendence of a director of husbandry. A locksmith's and joiner's shops give work to a certain number of the pensioners; the tailors' shop also occupies several patients. Side by side with these workshops is that of basket-making and chair-mending, which afford a pleasant and useful change to the other epileptics. Amongst the trades that could be easily and safely taught, I would place coopering and shoemaking. All these trades, in fact, can be carried on in any place, and, except those of the locksmith and carpenter, do not demand any great initial outlay; besides which, they have the advantage that they may be carried on at home, and do not necessitate continuing in a workshop amongst healthy workmen, to whom the sufferers may be objects of repulsion. The rule is that an epileptic does not remain in a workshop where he has had several seizures.* The master is afraid of accidents to his ailing workman, for which he might be held responsible. The seizures alarm, or at least disturb the other workmen, and the poor epileptic, turned away like a pariah, wanders from workshop to workshop, and remains in none. When he can work at home it is quite different; he works by the piece, or on his own account, can take care of himself, or be cared for during and after his seizures. He can earn an honest livelihood. This is the case with men; it is not quite the same with women. Beyond field labour, which is as well fitted for epileptic women as men, there exists scarcely any occupation for them except sewing and knitting. Some of our women and girl inmates work in the gardens and fields near the asylums, but the greater number are employed in workrooms, where, under the superintendence of a sewing mistress, they learn to cut out, to sew, to knit, and to embroider, in a word, to practise all kinds of needlework. This work being much too sedentary for sufferers from this disease, it is well to vary it as much as possible, and to divide it by other work needing less intensity of thought and more exertion of physical strength, therefore we employ our patients in all the other work of the house, but above all, in washing. Great care and constant supervision are necessary to prevent their falling into the water. Such are the occupations of the epileptic patients at the Asylum of Laforce."

Among the speakers who followed were Dr. Langdon Down, Dr. Buzzard, Dr. Rayner of Hanwell, Dr. Oliver, and others. We will note briefly the opinions of some of these eminent authorities, amongst others those of Dr. Langdon Down, who said, that he too had visited the Asiles John Bost with much interest, but that there was no attempt to make the employment remunerative. He thought that this should be attempted. Dr. Cobbold offered various practical suggestions as to the care that would have to exercised in admitting the workers, and considered that "all payments should be by piece-work, or in accordance with work done, and not by time." Miss Twining, a member of the Committee of the Society for Promoting Trained Nurses in Workhouses, bore witness to the usefulness of the work done in the wards of the Kensington Workhouse by epileptics, where they are employed according to their capacities. Miss Twining expressed great sympathy with the class of epileptics above the paupers, and spoke of the regret with which she had relinquished a Home she had conducted for sixteen years in Queen Square for a class who could pay a small sum, ten shillings a week. She said that there was no kind of needlework they could not undertake. Dr. Rayner of Hanwell thought epileptics might be employed in every way possible—shoemaking, tailoring, farming, provided there were careful supervision; but *before* they were so employed, they would have to be watched to see how far they could be trusted. Outdoor life was the best for them. The disease was in a great measure the product of life in towns, and was therefore infrequent in country districts. Any establishment should be placed in the country.

**Views of Eminent Authorities.**

The foregoing testimonies were all given with regard to the patients whose disease had reached that stage which requires active medical supervision, and it is therefore satisfactory to see that Dr. Buzzard, of the National Hospital for the Paralysed and Epileptic in Queen Square, speaks more hopefully than any of the previous witnesses. He says he has seen a great deal of the hardships entailed on epileptics by their inability to obtain employment. Employment was of great advantage to the patient himself. He would not enter into the question of what occupations were desirable, but, speaking from a medical point of view, he could say there were a large number of cases in which it would be comparatively easy to entrust the patient with the tools of his work.

**A Hopeful View.**

On the question of the desirability of employment for epileptics, it will be seen that all the doctors cordially agreed. A scheme was suggested at this meeting that would in some degree combine the requirements of the doctors, namely, work, supervision, fresh air, and continued treatment. A market garden presents many advantages as a field for employment, particularly if combined with the cultivation of bees and flowers. There would be much that would be suitable work in these for women also, and still the separation of the sexes might be carried out. Whether gardening or needlework be chosen; if an establishment be founded either for men or women (we cannot help hoping that the choice where needful, would fall on the weaker side), the medical aspects of the case would still be the same. What is required is that due

**A Practical Scheme.**

* This is speaking of France.

supervision be exercised, and accurate records be kept of each case. On the application of any worker for employment, a doctor's certificate would be required, with the history of the case to the date of application. Were market gardening chosen it would have the advantage that the workers could raise much of the food needed, as vegetable diet is often highly recommended. Wholesome work, due supervision by trained and competent persons of either sex, the removal, in case of need, to permanent medical care—these conditions would satisfy the medical side of the question; but there is still another aspect—the scheme must pay; at least, it must pay its expenses and support its *employés*. If the Union is relieved of those who would otherwise come upon it for support, it would but be fair that the bare allowance for the maintenance of these cases should be contributed. The boarding-house might certainly be made self-supporting, and those whose means permitted might be allowed to pay for better rooms, and so to contribute towards the medical expenses of the establishment, which would consist mainly in the supervision so strictly enjoined. Capital would have to be raised, and very little interest could be promised, but we do not believe that money would prove an insuperable difficulty. At first much gratuitous help might be needed in carrying out the scheme here outlined; but when the first needful mistakes inherent in all new ideas have been made and retrieved, we think and believe that it would pay, at least, for the maintenance of its workers, and give to them useful if hard-working lives, and above all save them from their present misery of being *involuntary paupers*.

The Scheme must Pay.

